# Ventricular Fibrillation following Varicella Zoster Myocarditis

**DOI:** 10.1155/2017/1017686

**Published:** 2017-11-23

**Authors:** Adam Ioannou, Irene Tsappa, Sofia Metaxa, Constantinos G. Missouris

**Affiliations:** ^1^Royal Free Hospital, London, UK; ^2^University of Cyprus Medical School, Nicosia, Cyprus; ^3^Frimley Health NHS Foundation Trust, London, UK

## Abstract

Varicella-zoster virus (VZV) infection can rarely lead to serious cardiac complications and life-threatening arrhythmias. We present a case of a 46-year-old male patient who developed VZV myocarditis and presented with recurrent syncopal episodes followed by a cardiac arrest. He had a further collapse eight years later, and cardiac magnetic resonance imaging (MRI) demonstrated mild mid-wall basal and inferolateral wall fibrosis. He was treated with an implantable cardioverter defibrillator (ICD) and represented two years later with ICD shocks, and interrogation of the device revealed ventricular fibrillation episodes. This case demonstrates the life-threatening long-term sequelae of VZV myocarditis in adults. We suggest that VZV myocarditis should be considered in all patients who present with a syncopal event after VZV infection. In these patients, ICD implantation is a potentially life-saving procedure.

## 1. Introduction

Infection with varicella-zoster virus (VZV) predominantly affects children and is, in most cases, a self-limiting and benign condition. However, in rare cases, it may lead to life-threatening cardiac complications [1]. We report a 46-year-old male patient who developed recurrent ventricular arrhythmias following the diagnosis of chicken pox.

## 2. Case Presentation

A 46-year-old male patient was first admitted to the emergency department of our hospital 12 years ago with recurrent episodes of collapse and a documented ventricular fibrillation (VF) arrest requiring emergency cardioversion by the paramedic team. The patient gave a 5-day history of general malaise and fever and a 24-hour history of an itchy vesicular rash. He had no relevant past medical history, but both his children had been diagnosed with chicken pox two weeks ago.

On examination, he was apyrexial, with a normal cardiovascular examination. He had multiple widespread erythematous, vesicular lesions approximately 2 mm across with some weeping that involved all limbs and his trunk. The haematological and biochemical investigations were normal, apart from a C-reactive protein (CRP) of 70 mg/L (normal ≤ 5 mg/L). The resting electrocardiogram (ECG) was within normal limits with a QTc interval of 403 msec. Transthoracic echocardiography confirmed normal biventricular structure and function with no regional wall motion abnormalities and normal cardiac valves. Intravenous amiodarone was administered for 24 hours, and no further arrhythmias were detected. In addition, he was treated with intravenous acyclovir for 10 days. He made an uneventful recovery. The patient took his own discharge a few days following the acute presentation.

**Figure 1:**
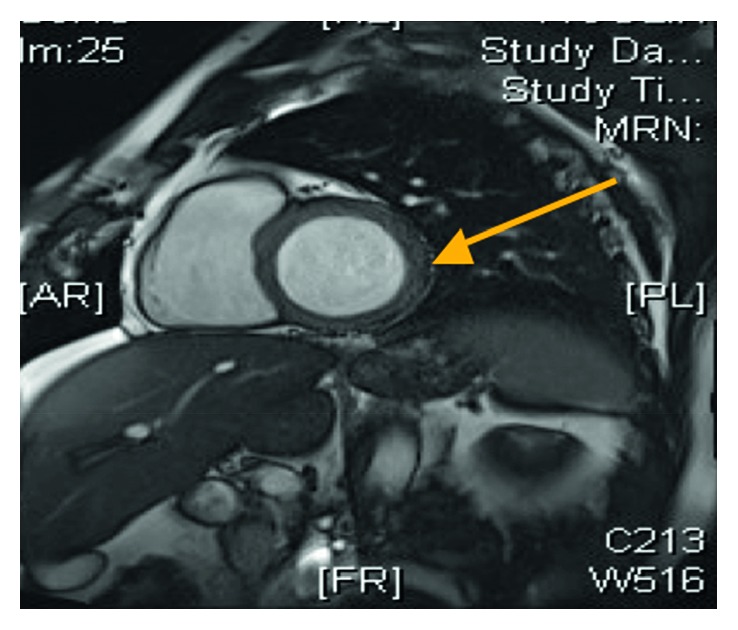
Short-axis view of the cardiac MRI demonstrating a normal left ventricular size and features consistent with myocarditis (arrow).

**Figure 2:**
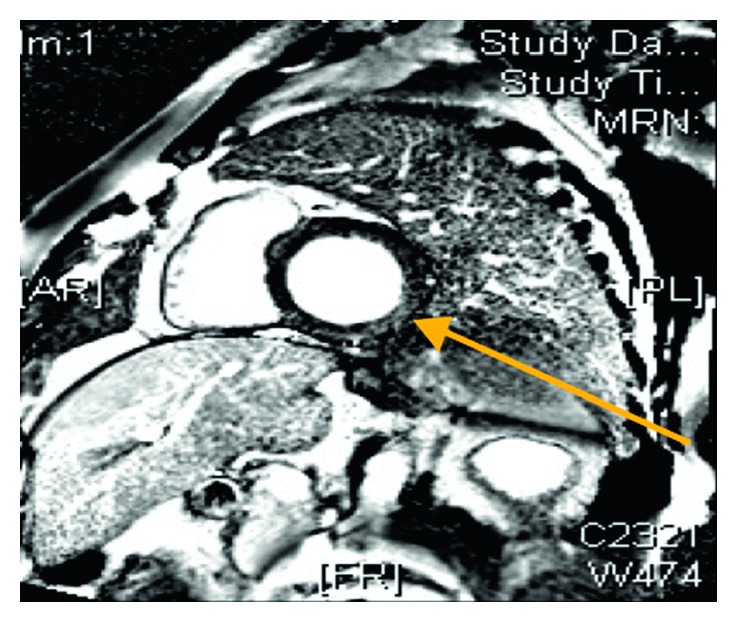
Cardiac MRI inversion recovery images after contrast injection revealing mild mid-wall enhancement in the basal inferior and inferolateral walls consistent with myocarditis (arrow).

He represented 8 years later with a further syncopal event lasting for less than a minute. He was on treatment with citalopram 20 mg od prescribed by his general practitioner for anxiety. He had a normal clinical examination, and the biochemical investigations were within normal limits. The resting ECG confirmed sinus rhythm with a normal QTc interval of 430 msec. The cardiac magnetic resonance imaging (MRI) confirmed normal biventricular function but also mild mid-wall myocardial enhancement at the basal inferior and inferolateral walls consistent with myocarditis and no evidence of inducible ischaemia (Figures 1 and 2). He was reviewed by the electrophysiology consultant, and no findings suggestive of channelopathy were identified, and the patient was treated with nebivolol 10 mg od and flecainide 100 mg bd. Following discussion in the multidisciplinary cardiology meeting, an implantable cardioverter defibrillator (ICD) was implanted (Boston Scientific ENERGEN F142).

**Figure 3:**
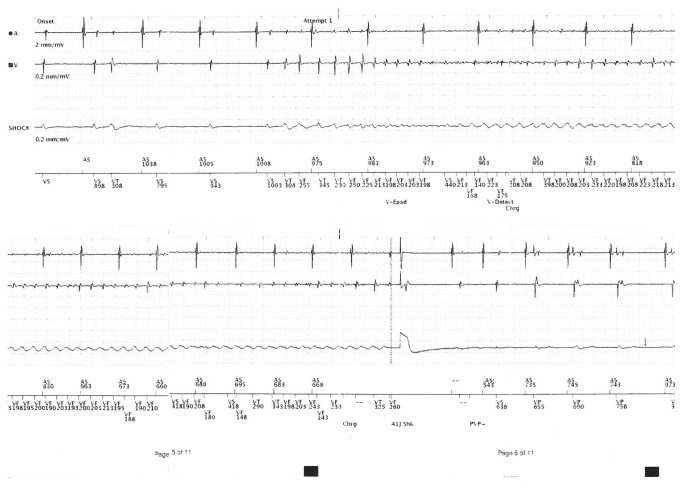
Interrogation of the dual-chamber defibrillator revealing an episode of ventricular fibrillation, followed by a shock.

Two years later, he developed 2 further episodes of VF leading to ICD activation and shock. On examination, his heart rate was 67 beats per minute and regular, and the supine blood pressure was 163/58 mm Hg. He had normal heart sounds and no signs of heart failure. The routine full blood count and biochemistry were normal (serum potassium 4.2 mmol/L and magnesium 0.76 mmol/L), and the high sensitivity troponin was normal (3 ng/L). ICD interrogation revealed two appropriate shocks for VF (Figure 3). The resting ECG confirmed a normal QTc duration of 420 msec. The transthoracic echocardiogram confirmed normal biventricular structure and function with an ejection fraction of 55–60%. There were no regional wall motion abnormalities, and the valves were structurally normal.

The patient continued to experience unifocal ventricular ectopic beats and short runs of nonsustained ventricular tachycardia (NSVT). As a result, flecainide was stopped, and he was treated with intravenous amiodarone. He made an uneventful recovery and was discharged home on nebivolol 10 mg od and amiodarone 200 mg od. Ablation therapy was not considered as an option as all the shocks were the result of VF and not VT.

## 3. Discussion

VZV infection leading to chicken pox is a common condition with the majority of cases occurring in childhood and is usually a benign and self-limiting disease. However, rarely, the infection may lead to life-threatening sequelae including encephalitis, myocarditis, and pneumonitis. These complications are more common in adults [1, 2].

More than 20 viruses have been shown to cause myocarditis in humans. Varicella myocarditis was first described in 1953, based upon a study of seven necropsy findings [3], and in 1977, Fiddler et al. [4] reported a 10-year-old child who developed syncopal events caused by VT and VF after contact with his grandfather who had a VZV infection 2 weeks earlier.

It is believed that the virus has a direct cytotoxic effect on the cardiac myocytes causing myocytolysis, necrosis, and oedema. In the acute phase, there is marked focal interstitial myocarditis with a collection of mononuclear cells, lymphocytes and occasional plasma cells, neutrophils, and eosinophils. Autoimmune reactions are also believed to take place. Following the acute inflammatory response, the resultant fibrosis and scarring leads to an electrical conduction block and reentry circuits predisposing patients to life-threatening ventricular arrhythmias. Furthermore, VZV myocarditis may mimic acute myocardial infarction, and in some patients, it may lead to congestive heart failure [4–6].

In these patients, anti-arrhythmic drugs such as flecainide, that acts on the sodium ion channels to delay myocyte recovery from excitation, also slows conduction through the scar tissue and may increase the risk of ventricular arrhythmias. Radiofrequency ablation treatment of scar tissue, resulting from myocarditis, is often challenging, as the scar tissue is often intramural or epicardial. In all these patients, the implantation of an ICD device is required to terminate the ventricular arrhythmia either by the delivery of a high voltage shock or a burst of rapid ventricular pacing to interrupt the reentry circuit. Treatment with the antiviral agent acyclovir is only beneficial in the early stages of viral replication within the myocardium, which coincides with the appearance of skin lesions [5, 7].

Our case clearly demonstrates that VZV infection in an adult patient may lead to life-threatening ventricular arrhythmias not only in the acute phase but long after the initial presentation. To our knowledge, this is the first case report of ventricular arrhythmias developing many years after the acute presentation of a VZV infection. We suggest, therefore, that in patients who present with a syncopal event after VZV infection, a high index of suspicion is required to investigate for potentially life-threatening ventricular arrhythmias. In these patients, the use of beta-blockade therapy and/or amiodarone, intravenous acyclovir, and ICD implantation is likely to improve the long-term outcome and prognosis.
